# Mild-temperature photothermal assisted CuSi nanowires for promoting infected wound healing

**DOI:** 10.3389/fbioe.2023.1158007

**Published:** 2023-03-03

**Authors:** Yanping Feng, Mingzhen Wu, Haidong Zhang, He Xu, Huili Li, Dongmin Chen, Hongyi Jiang, Jiang Chang, Zhihong Dong, Chen Yang

**Affiliations:** ^1^ College of Mechanical Engineering, Chengdu University, Chengdu, Sichuan, China; ^2^ Joint Centre of Translational Medicine, the First Affiliated Hospital of Wenzhou Medical University, Wenzhou, China; ^3^ Zhejiang Engineering Research Center for Tissue Repair Materials, Wenzhou Institute, University of Chinese Academy of Sciences, Wenzhou, China; ^4^ College of Chemistry and Materials Sciences, Shanghai Normal University, Shanghai, China; ^5^ Affiliated Hospital of Nanjing University of Chinese Medicine, Jiangsu Province Hospital of Chinese Medicine, Nanjing, China

**Keywords:** nanowire, anti-bacteria, angiogenesis, infected wound, photothermal therapy

## Abstract

In clinical practice, the utilization of antibiotics is still the main approach for the treatment of wound contamination, which lacks the ability to accelerate wound healing and arises the global concern of antimicrobial resistance. Plenty of alternative methods have been explored in recent years due to the fast development of material science. Here, CuO/SiO_2_ nanowires (CuSi NWs) with good near-infrared (NIR) photothermal conversion ability are synthesized by a one-step hydrothermal method. The as-prepared CuSi NWs possess excellent antibacterial ability against both *Escherichia coli* (*E. coli*) and *Staphylococcus aureus* (*S. aureus*), which could be enhanced by the assistance of mild photothermal therapy (PTT). Moreover, CuSi NWs at suitable concentrations can promote proliferation, migration, and angiogenic gene expression of human umbilical vein endothelial cells (HUVECs), exhibiting a remarkable pro-vascularization ability. The *in vivo* mouse infect model further proves that the CuSi NWs might be a good candidate for the treatment of infected wounds as the high antibacterial efficiency and accelerated wound healing is obtained.

## 1 Introduction

Treatment of bacterial infection is always an important part in the management of cutaneous wound healing. Severe infection can not only hinder wound healing progress, but also invade deep tissues, causing organ failure or even death (Zilberman and Elsner, 2008; Mei et al., 2021; Zeng et al., 2022). Although antibiotics have been proven as effective methods to deal with bacterial infection for centuries in the clinic, the following drug resistance is becoming a major health and medical crisis (Yu et al., 2018; Bakkeren et al., 2020). On the other hand, even if antibiotics can effectively kill bacteria, they cannot fundamentally accelerate the healing process of the wound, leading to the possibility of secondary infection and the chance to turn acute wounds into chronic wounds. Therefore, a treatment that can both combat bacterial infection and promote wound closure is urgently needed to be developed.

In recent years, non-invasive photothermal therapy (PTT) has been acknowledged as one of the primary alternatives to antibiotics (Doherty et al., 2010; Chen et al., 2020). The principle of PTT is converting light into heat by utilizing photothermal agents to destroy the integrity of pathogenic bacteria while avoiding bacterial resistance (Xu et al., 2019; Huo et al., 2021). The efficiency of photothermal antibacterial is directly related to the temperature, and a high temperature of more than 50°C is usually required for achieving a high antibacterial rate. For example, after NIR irradiation, the temperature of infected sites increased to 56°C which was much higher than surrounding normal skin, and showed excellent antibacterial activity against methicillin-resistant *S. aureus* (MRSA) (Qian et al., 2018). However, the application of hyperthermia may also cause damage to normal tissues, and even delay wound healing, making PTT hard be implemented alone (Xu et al., 2018; Huo et al., 2021; Bai et al., 2023). Therefore, the combination of PPT with other antibacterial treatment methods to achieve high antibacterial efficiency under mild temperatures is becoming the focus of photothermal antibacterial therapy.

Metal ions such as silver (Ag) and copper (Cu) ions are widely used in different antibacterial scenarios including textile fabrics, water treatment, and biomedical applications due to their broad-spectrum antibacterial properties (Chernousova and Epple, 2013; Slavin et al., 2017). For example, Ag ions containing wound dressing are proven effective for multiple microorganisms such as *Staphylococcus aureus* (*S. aureus*), *Escherichia coli* (*E. coli*), and *Candida albicans* (*C. albicans*) (Panacek et al., 2006; Stanic et al., 2011). However, its high cytotoxicity and potential biosafety risks are concerning (Rai et al., 2009; Perdikaki et al., 2016). In contrast, Cu ions showed less antibacterial efficiency but higher biocompatibility. As one of the trace elements of the human body, Cu is essential to maintain homeostasis and the physiological functions of various enzymes such as lysyl oxidase (Das et al., 2015; Das et al., 2016). Moreover, copper ions at low concentrations (0.064–6.4 ppm) can promote endothelial cell proliferation, migration, and vascularization, thus accelerating wound healing (Wu et al., 2013; Kong et al., 2014; Li et al., 2016). However, low concentrations of copper ions have limited antibacterial effects, and combination with other antibacterial means is usually required to achieve the ideal antibacterial therapeutic effect (Ning et al., 2015; Li t al., 2019). For example, Xu Qing et al. developed a PDA-modified Cu-doped mesoporous silica (Cu-MSN), which produced remote “hot ions” effect under the near-infrared (NIR) light and isplayed highly efficient, quick, and long-term inhibition of bacterial pathogens methicillin-resistant *S. aureus* (MRSA) and *E. coli* as well as bacterial biofilm (Xu et al., 2020). More interestingly, the sustained release of Cu and Si ions promoted wound closure by enhancing angiogenesis during the infectious wound healing process. However, the amount of doped Cu in Cu-MSN is limited, which may affect the controllability of Cu ion release. Also, additional photothermal agents such as PDA are required in the above system due to the lack of photothermal covert ability of Cu-MSN, which greatly increases the uncertainty of its practical application.

**FIGURE 1:**
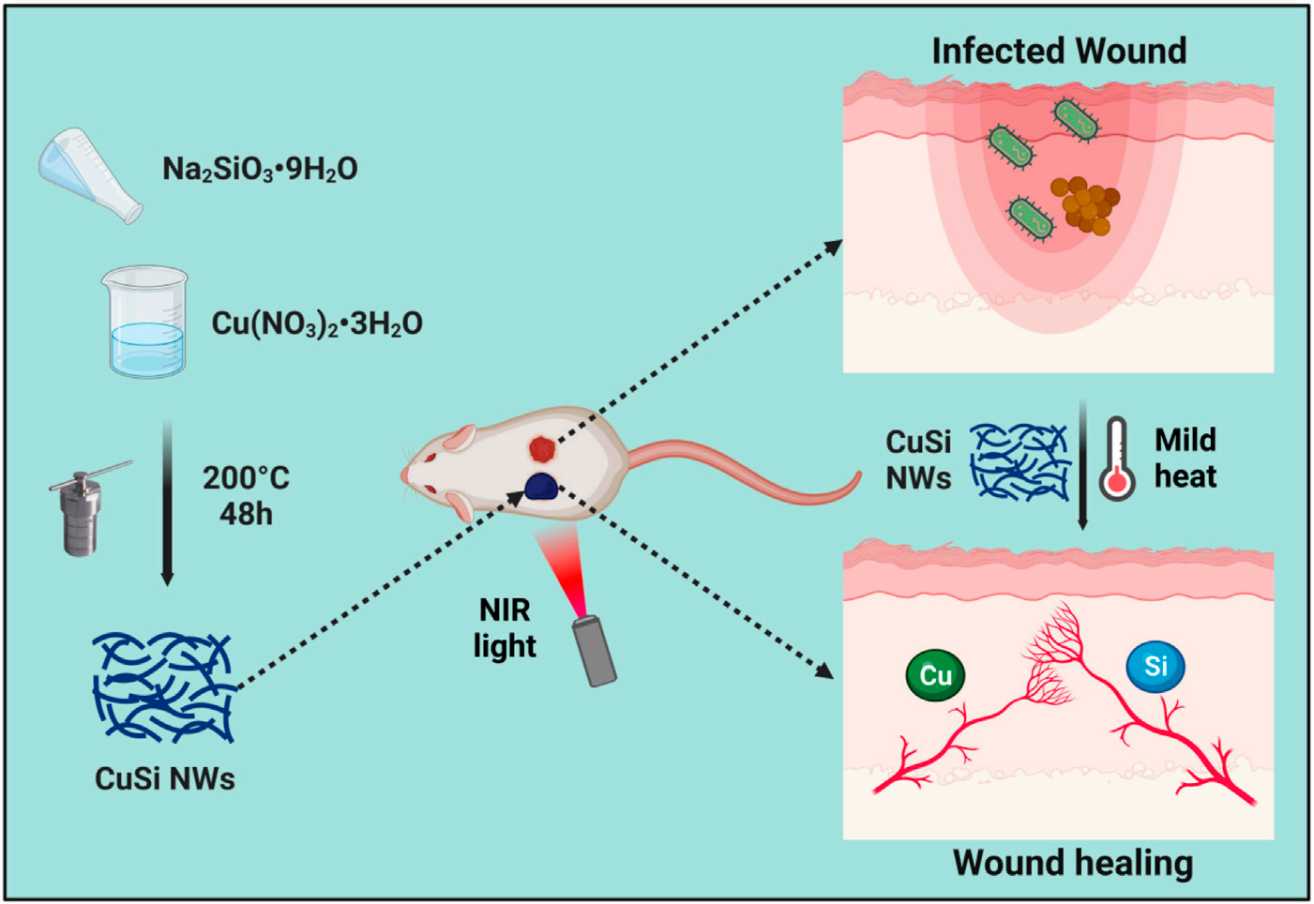
CuSi NWs with light-to-heat conversion capability was designed to effectively combat bacteria and simultaneously promote angiogenesis, thereby improving the wound healing rate of infected wounds. The figure was created with BioRender.com.

Herein, we synthesized a copper oxide/silica (CuSi) mixed nanowire (NW) with inherent NIR photothermal properties. The antibacterial effect of CuSi nanowires under NIR light stimulation on both *E.coli* and *S. aureus* was investigated. Meanwhile, the effects of CuSi nanowires on endothelial cell proliferation, migration, and pro-vascularization were studied. Finally, a mouse-infected wound model was established to evaluate the therapeutic effect of CuSi nanowires on promoting infected wound healing. The study provides a new strategy for the treatment of infected wounds. Figure 1.

## 2 Materials and methods

### 2.1 Materials

All chemical reagents were from Aladdin Biochemical Technology Co., Ltd. (Shanghai, China) except those otherwise mentioned.

### 2.2 Preparation of CuSi NWs

CuSi NWs were synthesized following a modified hydrothermal method (Ma et al., 2022). Briefly, 20 mL sodium metasilicate non-ahydrate (Na_2_SiO_3_·9H_2_O, 0.2 M) and 20 mL copper nitrate trihydrate (Cu(NO_3_)·3H_2_O, 0.2 M) aqueous solution were mixed evenly into a polyphenylene lined stainless steel autoclave and hydrothermally treated at 200°C for 48 h. Then, the precipitation was collected by centrifugation at 10,000 rpm/min for 5 min. The as-obtained CuSi NWs were rinsed three times with Milli-Q water and freeze-dried in a lyophilizer (SCIENT2-10N/A, Ningbo Xinzhi Biotechnology Co., Ltd., China) for further use.

### 2.3 Characterization of CuSi NWs

The morphologies of CuSi NWs were observed using a scanning electron microscope (SEM, SU8010, HITACHI, Japan) and the analysis of elements by mapping of energy dispersive spectrometry (EDS) were detected by Phenom scanning electron microscope (Phenom Pharos, Phenom, Netherlands). The X-Ray Diffraction (XRD) patterns of CuSi NWs were collected using an X-ray diffractometer (D8 ADVANCE, Bruker, Germany). Fourier transform infrared (FTIR) spectra of CuSi NWs were measured by an FTIR spectrometer (Tensor II, Bruker, Germany). The ultraviolet-visible-near-infrared (UV-VIS-NIR) absorption spectra of CuSi NWs were tested using a spectrometer (CARY5000, Agilent, United States). To evaluate the ions release concentration of CuSi NWs, CuSi NWs were dispersed in Milli-Q water with weight-to-volume concentrations of 31.25 μg/mL. The aqueous dispersion was diluted, filtrated and collected at different days, then detected by inductively coupled plasma mass spectrometry (ICP).

### 2.4 Photothermal performance characterization of CuSi NWs

To evaluate the photothermal properties of CuSi NWs, CuSi NWs were dispersed in Milli-Q water with weight-to-volume concentrations of 0.5, 1, 2, 4, and 8 mg/mL, respectively. The samples were then irradiated with an 808 nm laser (Ningbo Yuanming Laser Technology Co., Ltd., China) using the power density of 1 W/cm^2,^ and the corresponding real-time temperature was recorded using an infrared thermal imager (A300, FLIR, United States). In addition, the real-time temperature of CuSi NWs aqueous dispersion (2 mg/mL) under different power densities (0.6, 1, and 1.4 W/cm^2^) was also recorded. Finally, to confirm the photothermal stability of the CuSi NWs, a heating cycle test was performed on the 2 mg/mL CuSi NWs dispersion solution. Briefly, the sample was irradiated by the laser at 808 nm using the power density of 1 W/cm^2^ for 5 min and then cooled in the air for another 5 min. The whole process was repeated 6 times.

### 2.5 Effects of CuSi NWs on viability of HUVECs

To evaluate the cytocompatibility of CuSi NWs, human umbilical vein endothelial cells (HUVECs) were purchased from the National Collection of Authenticated Cell Cultures (Shanghai, China) and cultured in Dulbecco’s modified eagle medium (DMEM) containing 10% (v/v) fetal bovine serum (FBS) and 1% penicillin/streptomycin (P/S). For cell viability assay, HUVECs were seeded in 96-well cell culture plates with a density of 1 × 10^3^ cells/well and incubated at 37°C with the supplement of 5% CO_2_ for 24 h. Subsequently, the media were replaced with 100 μL fresh media containing different concentrations of CuSi NWs (125, 62.5, 31.25, 15.625, and 7.8125 μg/mL) to further treat cells for 1, 2, and 3 days, respectively. The CuSi NWs-free culture medium was used as a control. A CCK8 assay kit (40203ES80, YEASEN, China) was then implemented for cell viability assay following the manufacturer’s instructions. Briefly, the media were replaced with CCK-8 solution, and the plates were put back into the incubator at 37°C for another 1 h, using a microplate reader (EPOCH2, BioTek, United States) to another 96-well plate for the test of light absorbance at 450 nm.

### 2.6 Effects of CuSi NWs on migration of HUVECs *in vitro*

HUVECs were seeded in 6-well plates and incubated at 37°C for 24 h. The seeding density of HUVECs was 4.5 × 10^5^ cells/well. Then, a 10 μL pipette tip was used to draw a straight line at the bottom of the plate, and the media were refreshed with DMEM containing 1% FBS and 1% P/S, or media containing CuSi NWs (31.25 μg/mL), respectively. Photographs of cells were taken by a microscope (SZ61TR, Olympus, Japan). After treating for another 24 h at 37°C, cells were fixed with 4% paraformaldehyde for 10 min and stained with 0.4% crystal violet for 5 min. Photographs of cells were taken again by the microscope and the cell migration rate was calculated using Image J software.

### 2.7 Effects of CuSi NWs on tube formation of HUVECs *in vitro*

First, 120 μL of Matrigel™ dilution was added to a 48-well plate and incubated at 37°C for 40 min. Then, HUVECs were seeded onto the well plate with a density of 4 × 10^4^ cells/well. DMEM containing 10% FBS and 1% P/S or media containing CuSi NWs (31.25 μg/mL) were used to treat HUVEC for 6 h at 37°C and the normal media were used as the Blank group. Finally, seven randomly selected areas were photographed by the microscope (SZ61TR, Olympus, Japan), and the tubular length and tube number were analyzed using Image J software.

### 2.8 The gene expression in HUVECs treated by CuSi NWs

The mRNA expressions of *VEGF*, *bFGF*, and *HIF-1α* in HUVECs were detected by quantitative polymerase chain reaction (qPCR). Briefly, HUVECs (3 × 10^5^ cells per well) were seeded on 6-well culture plates and cultured for 24 h. Then, the cells were treated with the above-mentioned media for other 3 days. After that, HUVECs were washed twice with preheated PBS and the mRNA was extracted from cells using an RNA extraction kit (M3211070, YEASEN, Shanghai, China). Subsequently, the extracted RNA was transcribed into cDNA using Hifair®Ш 1st Strand cDNA Synthesis SuperMix for qPCR (gDNA digester plus) (H6201080, YEASEN, Shanghai, China). PCR amplification was further implemented with primers of *VEGF*, *bFGF*, *HIF-1α*, and the housekeeping gene *GAPDH*, which were synthesized by the company of Shenggong, Co., Ltd. (Shanghai, China). The sequences of primers were as Table 1.

**TABLE 1:** Primers for gene expression analysis.

Target gene	Forward primer sequence (5′-3′)	Reverse primer sequence (5′-3′)
*GAPDH*	GAT​TTG​GTC​GTA​TTG​GGC​G	CTGGAAGATGGTGATGG
*bFGF*	CAA​TTC​CCA​TGT​GCT​GTG​AC	ACC​TTG​ACC​TCT​CAG​CCT​CA
*VEGF*	GAA​GAA​AGT​GGT​GCC​ATG​GAT​AG	CCC​ATG​AGT​TCC​ATG​CTC​AGA
*HIF-α*	CAA​CGT​GGA​AGG​TGC​TTC​A	CGG​CTC​ATA​ACC​CAT​CAA​CT

### 2.9 Antibacterial ability of CuSi NWs *in vitro*

*Staphylococcus aureus* (*S*.*aureus*) and *Escherichia coli* (E.*coli*) were used for anti-bacterial experiments. First, *S.aureus* and *E. coli* were cultured with LB broth in a shaker at 37°C for 4 h, and then diluted with saline to the density of 1 × 10^6^ CFU/mL. Next, 100 μL saline or saline containing CuSi NWs (62.5 μg/mL) was mixed into 100 μL of bacterial solution. Then for CuSi + NIR group, the obtained samples were irradiated with an 808 nm laser for 15 min and the terminal temperature was maintained at ∼ 45°C (monitored by the infrared thermal imager). Then, after incubating the *S.aureus* and *E.coli* solution for another 4 h, the number of bacteria was calculated using the plating method.

### 2.10 Therapeutic effect of CuSi NWs on infected wounds

Male ICR mice (8-week-old, 30–35 g) were obtained from Zhejiang Animal Center (Zhejiang, China). The animal protocol and experimental procedures were approved by the Animal Research and Ethics Committee of Wenzhou Institute of the University of Chinese Academy of Sciences (Approval Issue No. WIUCAS22102007). A mouse model of infected wounds was established as follows: First, the mice were anesthetized with 1.5% sodium pentobarbital. Then, the hair on the backs was shaved and the nude backs were wiped with iodine. Next, two circular full-thickness wounds on the upper back of the mice were created using a sterilized hole punch (8 mm diameter), and 20 μL *S.aureus* suspension (1 × 10^7^ CFU/mL) was added to the wounds to create infectious wounds. The mice were randomly divided into three groups and received different treatments, namely, Blank, CuSi, and CuSi + NIR. For Blank group, 50 μL of PBS solution was applied. For CuSi and CuSi + NIR groups, 50 μL of PBS solution containing CuSi NWs (31.25 μg/mL) was dropped on the infected wound each time, while in the CuSi + NIR group, an 808 nm laser was applied for the photothermal therapy in the first 3 days immediately after the dropping of CuSi NWs suspension. The average temperature of the infected wound irradiated by laser was monitored by the infrared thermal imager and maintained at ∼45°C for 15 min. Photographs of wounds were taken at selected time points (Day 0, Day 3, Day 6, and Day 9). In addition, on day 3, the pus from the wound was taken with a disposable sterile cotton swab and transferred into 2 mL of saline. After shaking vigorously, 20 μL of the samples were taken and dispersed on an agar plate for evaluation of bacterial infection of the wound site. On day 9, all mice were sacrificed to obtain skin samples.

### 2.11 Histological analysis

The obtained tissue samples were fixed in 4% paraformaldehyde for 24 h, then washed with current water, dehydrated by graded ethanol, embedded by paraffin, and cut into 5 μm sections for hematoxylin-eosin (H&E), Masson, and CD31 immunohistochemical staining. For all staining methods, sections were dewaxed and hydrated first. For H&E staining, sections were stained with hematoxylin and eosin following the manufacturer’s instructions (H&E, Sigma-Aldrich). After being captured by microscope (KF-PRO-005, KFBIO, China), the lengths of the wounds were measured using ImageJ software. For Masson staining, sections were stained with Ponceau S and Aniline Blue following the manufacturer’s instructions. After being captured by the microscope, collagen deposition fractions were calculated using Image-Pro Plus software. For immunohistochemical staining, sections were antigen retrieved and antibody anti-CD31 (bs-0196R, Bioss, Beijing, China) was used. The number of blood vessels counted based on the CD31 staining.

### 2.12 Statistical analysis

All results were expressed as means ± standard error of mean. Multiple comparisons between groups were performed using one-way ANOVA testing with a *post hoc* test. Statistical significance was considered when *p < 0.05 or **p < 0.01 or ***p < 0.001 (^#^ *p* < 0.05 or ^##^ *p* < 0.01 or ^###^ *p* < 0.001).

## 3 Results

### 3.1 Characterization of CuSi NWs

**FIGURE 2:**
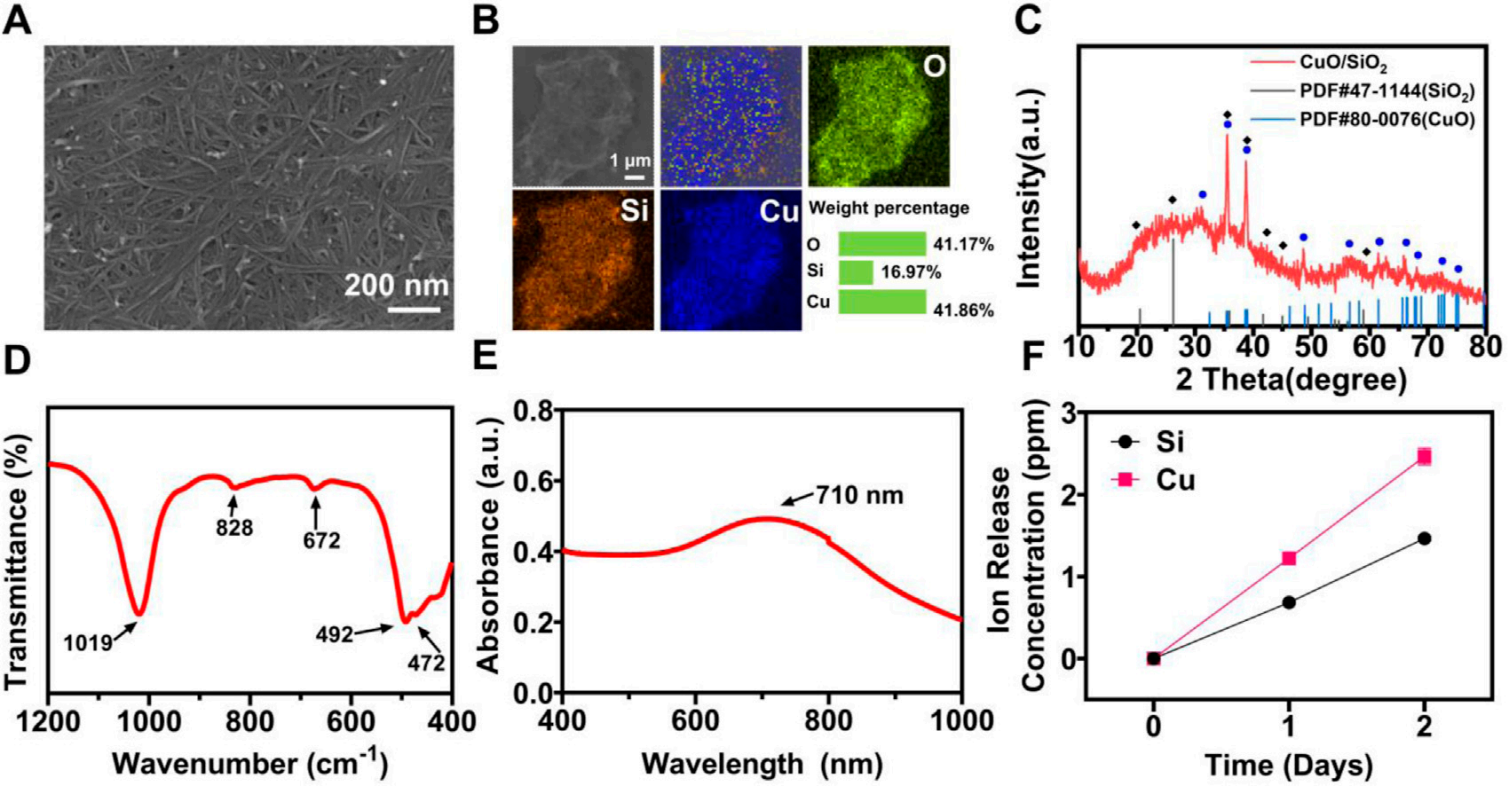
Characterization of CuSi NWs. **(A)** SEM image. **(B)** EDS mapping. **(C)** XRD pattern. **(D)** FTIR spectra. **(E)** UV-VIS-NIR spectrograms. **(F)** Accumulated release of Si and Cu ions from CuSi NWs at different days.

CuSi NWs were prepared by a modified hydrothermal reaction method. It can be clearly observed from the scanning electron microscope (SEM) image that the diameter of CuSi NWs was about 10 nm and the length reached about 600 nm (Figure 2A). The energy dispersive spectrometry (EDS) element mapping showed that Cu, Si, and O elements were uniformly distributed in CuSi NWs (Figure 2B). The obtained CuSi NWs were then evaluated by X-ray diffraction (XRD), Fourier-transform infrared spectroscopy (FTIR), and ultraviolet-visible-near-infrared (UV-VIS-NIR) spectrophotometer. It can be found from the XRD pattern that CuSi NWs were mainly constituted by the phase of CuO and SiO_2_ (Figure 2C), which was further confirmed by FTIR analysis as the absorption peaks at approximately 1,019, 828, and 472 cm^−1^ were assigned to the different vibration modes of the Si–O–Si or O–Si–O bonds of amorphous SiO_2_, while peaks centered at 492 and 672 cm^−1^ indicate cm^−1^ Cu (II)–O species (Lefez et al., 1995; Li et al., 2019) (Figure 2D). In addition, the UV-VIS-NIR absorption spectra showed that CuSi NWs had a broad absorption from the wavelength of 400 nm to 1,000 nm, and a peak at 710 nm was observed (Figure 2E). As shown in Figure 2F, Si ions and Cu ions release concentration from CuSi NWs were about 0.685 ppb and 1.223 ppb per day, respectively.

### 3.2 Photothermal performance of CuSi NWs

**FIGURE 3:**
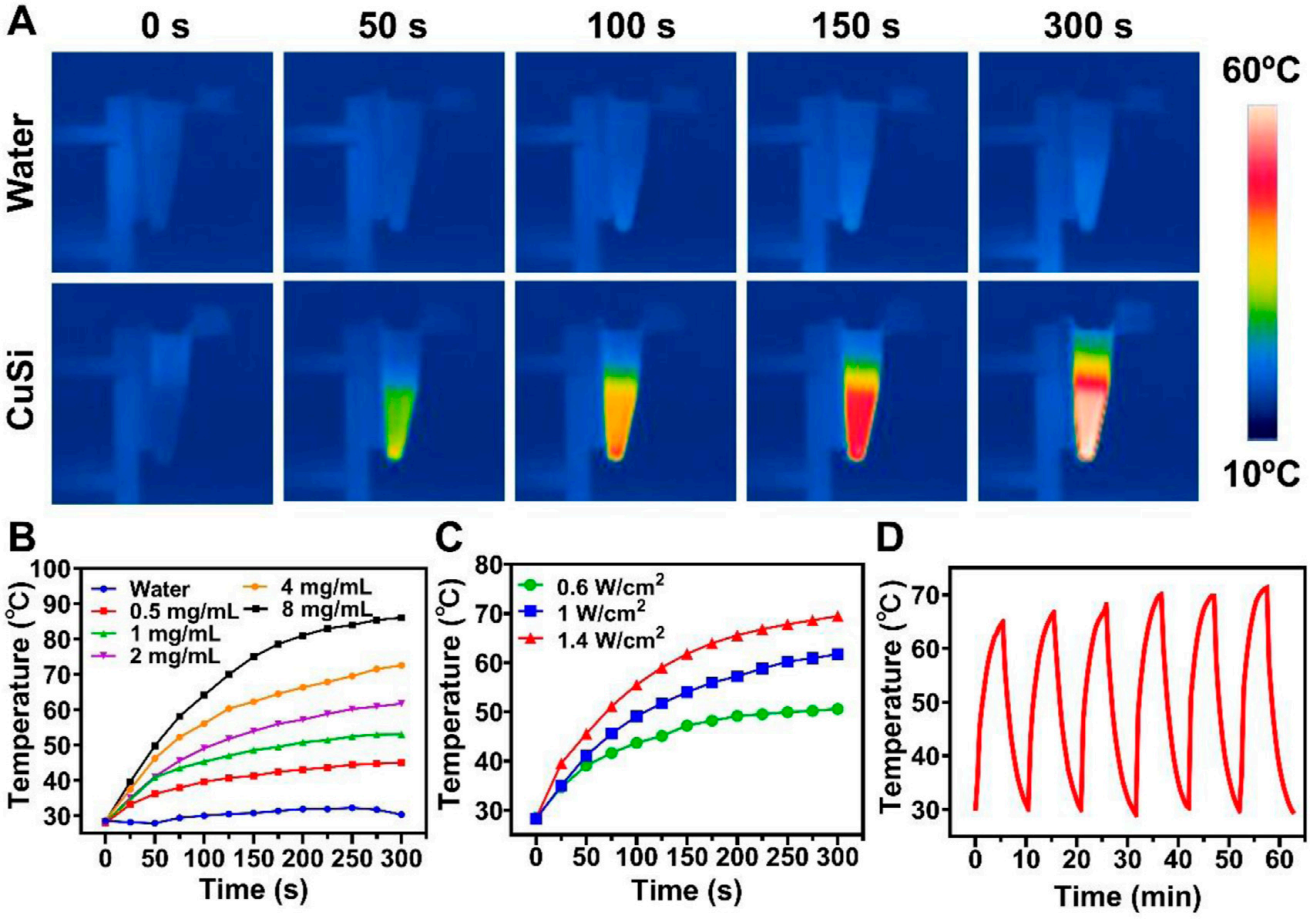
Light-to-heat conversion capability of CuSi NWs. **(A)** Representative thermal images of water and CuSi NWs aqueous dispersions under the 808 nm laser irradiation. **(B)** Photothermal heating (1 W/cm^2^) curves of different CuSi NWs aqueous dispersions for 5 min. **(C)** Photothermal heating curves of CuSi NWs aqueous dispersion (2 mg/mL) under 808 nm laser irradiation at varying power densities (0.6, 1, and 1.4 W/cm^2^). **(D)** Photothermal stability of CuSi NWs aqueous dispersion (six laser on/off cycles).

The photothermal performances of CuSi NWs were evaluated under 808 nm laser irradiation. As shown in Figure 3A, the temperature CuSi NWs dispersion (2 mg/mL) rapidly increased from 28.2°C to 61.7°C in 5 min with light irradiation (1 W/cm^2^). In contrast, there is no evident temperature increment in pure water with the same treatment. The effect of the CuSi NWs aqueous dispersion concentration and laser power on photothermal performances were further investigated (Figures 3B, C). As expected, the temperature of the dispersion was positively correlated with the concentration of CuSi NWs aqueous dispersion or the power density of the laser. Specially, the maximum temperature of CuSi NWs aqueous dispersion at the concentration of 8 mg/mL could reach 86°C under 5 min’ laser irradiation (1 W/cm^2^). Moreover, the photothermal stability of CuSi NWs was verified as no particular change in the photothermal effect was noticed after six repetitive laser on/off cycles (Figure 3D).

### 3.3 Effect of CuSi NWs on proliferation, migration, and pro-vascularization of HUVECs

**FIGURE 4:**
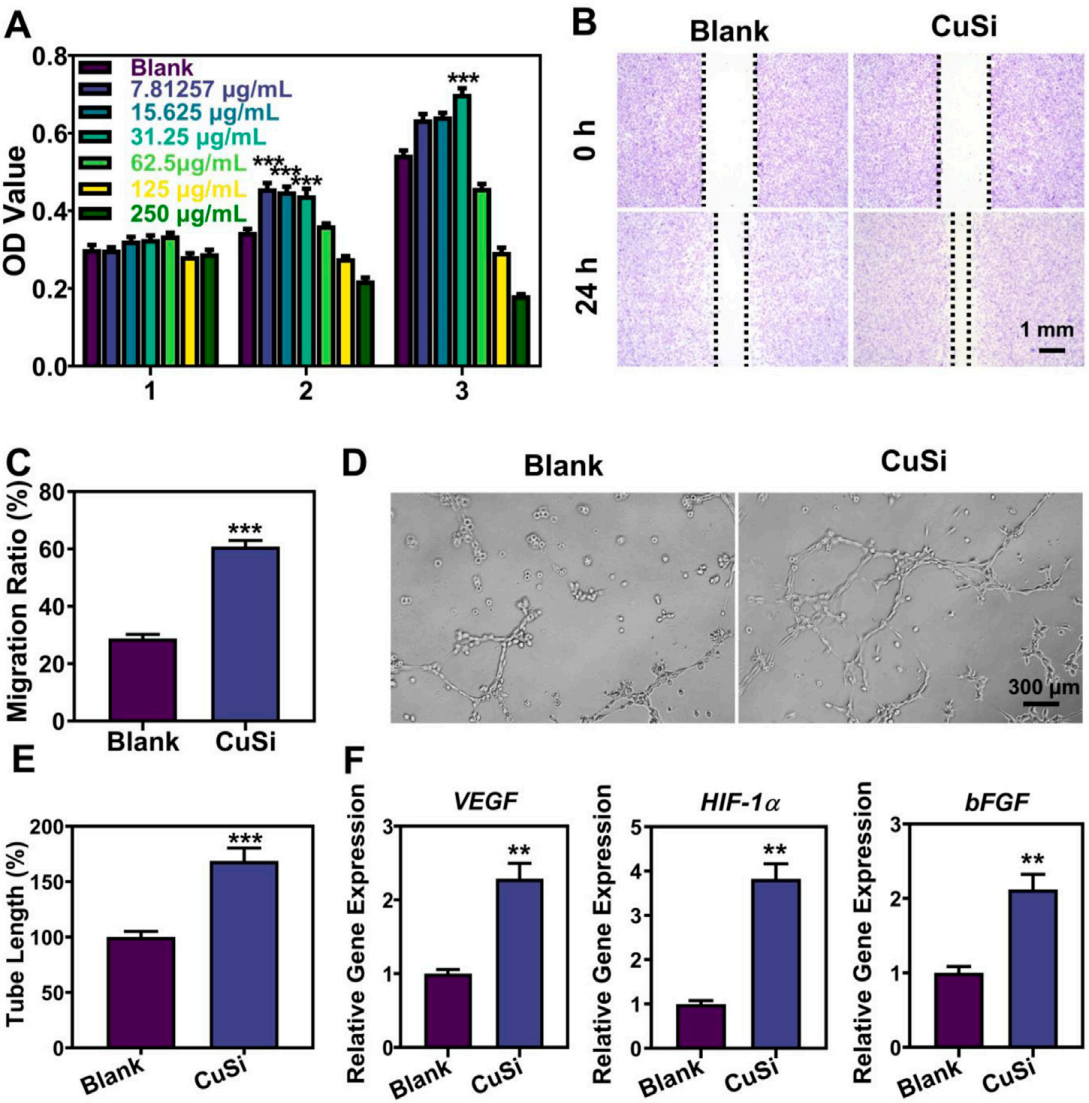
Effects of CuSi NWs on the proliferation, migration, and pro-angiogenesis of HUVECs. **(A)** Cell viability of HUVEC after treatment with different CuSi NWs concentrations (*n* = 6). **(B)** Representative cell migration photos after treatment with/without CuSi NWs for 24 h. **(C)** Quantification of cell migration rate (*n* = 8). **(D)** Representative newly formed tubes after treatment with/without CuSi NWs for 6 h. **(E)** Quantification of tube length (*n* = 7). The Blank group was normalized. **(F)** Angiogenic genes (*VEGF*, *HIF-1α* and *bFGF*) expression in HUVECs after treatment with CuSi for 3 days (*n* = 3). *p < 0.1, **p < 0.01 or ***p < 0.001.

Human umbilical vein endothelial cells (HUVECs) were used to evaluate the bioactivity of CuSi NWs. Firstly, the activities of HUVECs treated with different concentrations of CuSi NWs (7.8125∼125 μg/mL) for 1, 2, and 3 days were evaluated using a CCK8 assay, respectively. As shown in Figure 4A, CuSi NWs at high concentrations (62.5 and 125 μg/mL) showed a suppression effect on HUVEC activity. However, CuSi NWs at concentrations of 7.8125, 15.625, and 31.25 μg/mL significantly promoted the proliferation of HUVECs as compared to the Blank group. Notably, CuSi NWs at concentrations of 31.25 μg/mL had the best stimulation on HUVEC activity among all the groups. Therefore, we chose 31.25 μg/mL as the representative group of CuSi NWs for further cell experiments.

The migration of HUVECs with/without the treatment of CuSi NWs was further conducted and the results are presented in Figures 4B, C. In contrast to the Blank group, CuSi NWs promoted faster migration of HUVECs with a rate increment of 32.06%. Furthermore, the CuSi NWs were proved to significantly stimulate more tube formation of HUVECs *in vitro*. As shown in Figure 4D, more formed vascular rings were observed after the treatment of CuSi NWs. The corresponding quantitative analysis further confirmed the conclusion as the tube length was increased by 68.72% compared with Blank group when the Blank group was normalized (Figure 4E). Moreover, the effect of CuSi NWs on activating the expression of pro-angiogenic genes in HUVECs was evaluated, which exhibited that after treating with CuSi NWs, significantly higher expression of *bFGF*, *VEGF*, and *HIF-1α* was observed in HUVECs as compared to that in Blank group, implying the pro-angiogenic ability of CuSi NWs (Figure 4F).

### 3.4 Antibacterial performance of CuSi NWs

**FIGURE 5:**
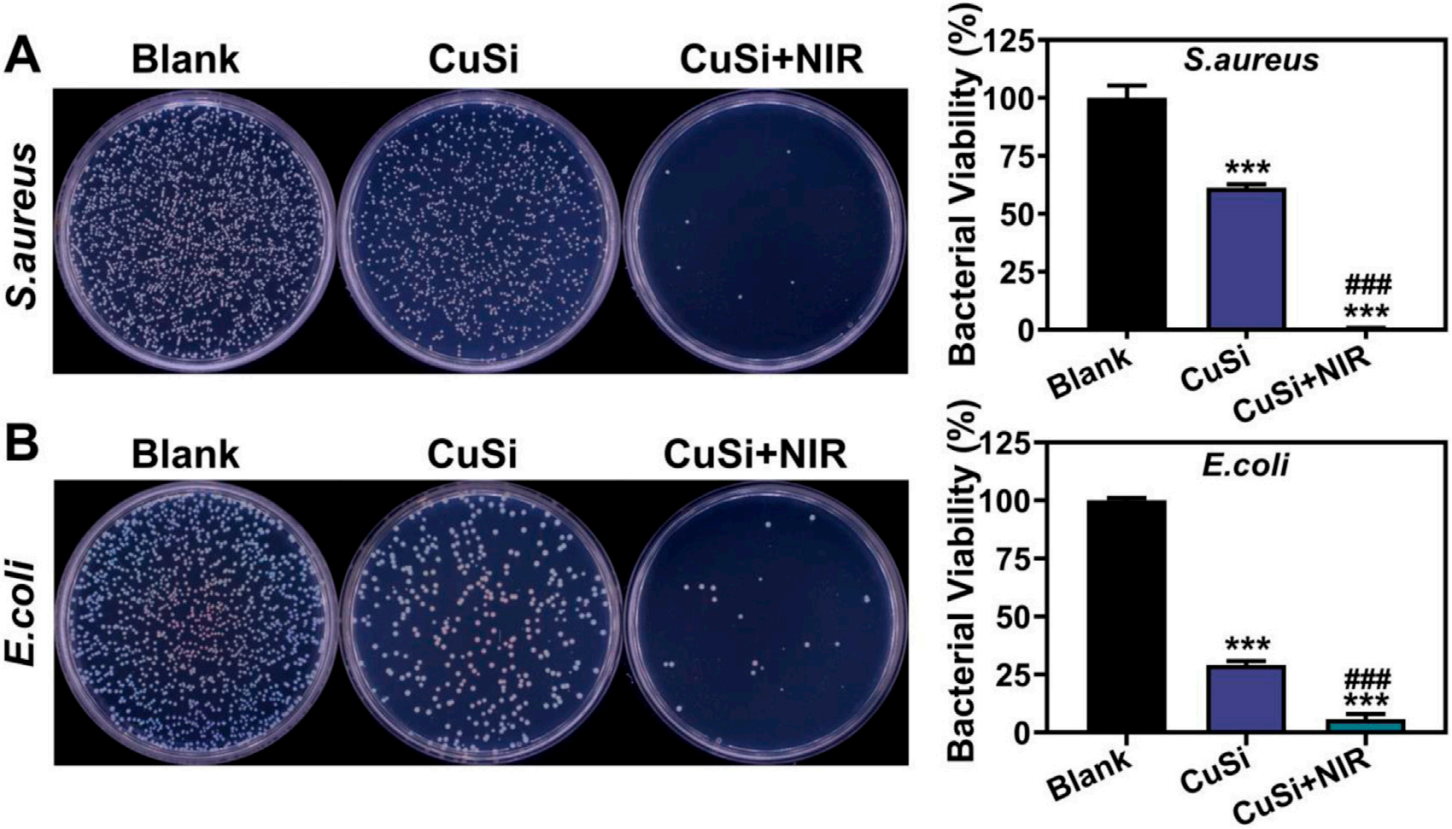
Antibacterial activity of CuSi NWs against *S. aureus* and *E. coli*. **(A)** Representative photos and bacterial viability percentage of *S. aureus* with different treatments (*n* = 3). **(B)** Representative photos and bacterial viability percentage of *E. coli* with different treatments (*n* = 3). The Blank group was normalized. *p < 0.1, **p < 0.01 or, ***p < 0.001 vs. Blank group; ^#^ *p* < 0.05 or ^##^ *p* < 0.01 or ^###^ *p* < 0.001 vs. CuSi group.

The antibacterial properties of CuSi NWs with/without NIR light irradiation were verified using both gram-positive (*S.aureus*) and gram-negative (*E.coli*) bacteria. As shown in Figures 5A, B, CuSi NWs alone had a broad antibacterial ability as they can suppress the growth of both *S.aureus* and *E.coli.* Such antibacterial performance could be further enhanced under the NIR light irradiation. The corresponding quantitative analysis displayed that the bacteria inhibitive rate of CuSi NWs alone were 38.69% ± 2.34% and 70.93% ± 3.22% against *S.aureus* and *E.coli*, respectively, which increased to 99.35% ± 0.16% and 94.19% ± 4.27% after the treatment of NIR light irradiation. The results proved that CuSi NWs and mild heat may produce a synergistic effect on killing bacteria.

### 3.5 Effect of CuSi NWs on infected wound healing

**FIGURE 6:**
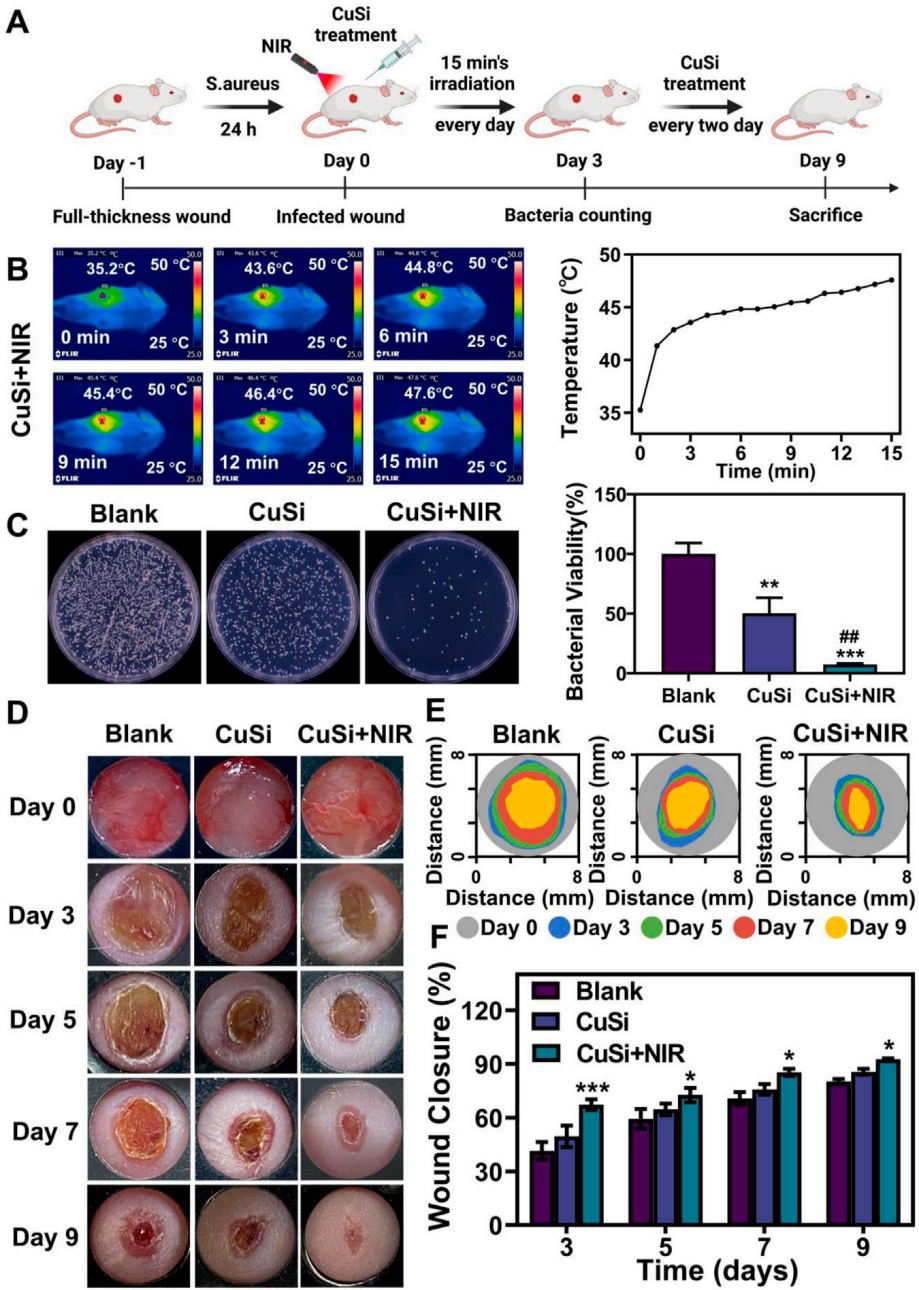
Treatment of infected wounds with CuSi NWs. **(A)** Schematic diagram of the protocol for the establishment of an infection model and the course of treatment. **(B)** Representative thermal images and the corresponding photothermal heating curves of the wound site treated with CuSi NWs under the irradiation of NIR light. **(C)** Representative photos and bacterial viability of *S. aureus* in wound site after different treatments (*n* = 6). The Blank group was normalized. **(D)** Representative photos, **(E)** size markers, and **(F)** quantitative closure rate of the wounds with different treatments on day 3, 5, 7, and 9, respectively (*n* = 6). Figure 5A was created with BioRender.com. *p < 0.1, **p < 0.01, or ***p < 0.001 vs. Blank group; ^#^ *p* < 0.05 or ^##^ *p* < 0.01 or ^###^ *p* < 0.001 vs. CuSi group.

To evaluate the photothermal therapeutic effect of CuSi NWs on the repair of infected wounds. We first established a mouse-infected wound model by the contamination of *S. aureus.* Three groups were implemented including Blank, CuSi, and CuSi + NIR. For the treatment of CuSi + NIR, the photothermal threatment was applied by using NIR light 15 min/day at the first 3 days, raised the temperature of wound to about 45°C, then add CuSi NWs suspension every 2 days afterwards (Figure 6A). As shown in Figure 6B, the temperature of the wound surface in CuSi + NIR group increased from 35.3°C to 47.6°C in 15 min. After treatment for 3 days, the residual bacteria in each group were collected for plate counting (Figure 6C). The bacterial viabilities in CuSi group and CuSi + NIR group were 50.36% ± 49.66% and 7.49% ± 3.49%, respectively, indicating the synergistic antibacterial effect of Cu ions and hyperthermia.

The wound healing process of each group was further displayed in Figures 6D, E. Both CuSi and CuSi + NIR could facilitate wound healing as compared to Blank group, while CuSi + NIR had the best stimulation effect among all groups. Through quantitative statistics (Figure 6F), it can be found that the wound areas in CuSi + NIR group were always the smallest among all groups at each time point, indicating the best wound healing ability of CuSi + NIR. Specifically, the wound closure rate in CuSi + NIR group on day 3, 5, 7, and 9 were 67.33% ± 10.26%, 72.68% ± 15.48%, 85.32% ± 6.9%, and 92.69% ± 2.14% respectively. Whereas, the corresponding wound closure rates were 41.49% ± 22.82%, 59.37% ± 18.57%, 70.76% ± 15.36%, and 80.21% ± 4.6% in Blank group, and 49.58% ± 18.41%, 64.65% ± 10.8%, 75.82% ± 9.7%, and 85.63% ± 5.76% in CuSi group.

**FIGURE 7:**
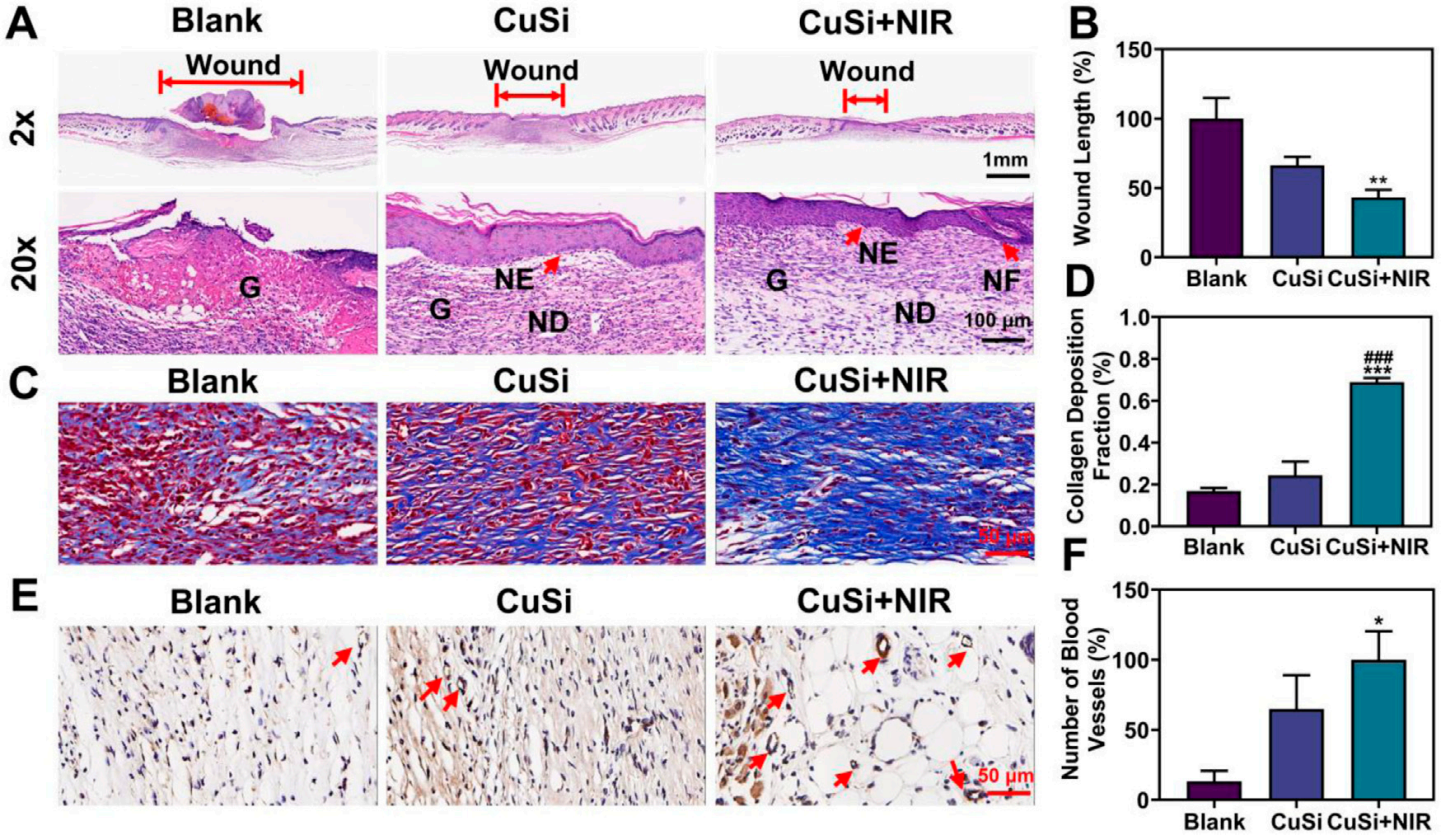
Histological analysis of wound sections after 9 days with different treatments. **(A)** H&E staining. The length of the red arrow represents the length of the unhealed wound bed (G: granulation tissue; NE: neoepidermis; ND: neodermis; HF: hair follicles). **(B)** Statistics of wound bed granulation length (*n* = 5). The Blank group was normalized. **(C)** Masson staining. **(D)** Statistics of collagen deposition distribution (*n* = 5). **(E)** Immunohistochemical staining pictures against CD31 and **(F)** the statistics of neovessel numbers (*n* = 5). The CuSi + NIR group was normalized in **(F)**. Arrows indicate blood vessels. *p < 0.1, **p < 0.01 or ***p < 0.001 vs. Blank group; ^#^ *p* < 0.05 or ^##^ *p* < 0.01 or ^###^ *p* < 0.001 vs. CuSi group.

In addition, histological analysis was performed to assess the quality of the repaired wounds with different treatments. Figures 7A, B show the H&E staining and corresponding quantification of the repaired wounds. Compared with the Blank group, both CuSi and CuSi + NIR stimulated less granulation tissue in the defect area, indicating better therapeutic effects on the maturation of granulation tissue, especially for CuSi + NIR group, in which the best dermal formation, epithelization, and hair follicle regeneration were observed. The Masson’s staining further revealed that both CuSi and CuSi + NIR promoted more collagen deposition in the wounds compared with the Blank group, while CuSi + NIR had the best collagen deposition outcomes among all groups (Figures 7C, D). Moreover, the newly formed vessels were evaluated by immunohistochemistry staining of CD31. As shown in Figures 7E, F, more neovascularization was observed in the CuSi + NIR group compared with both Blank and CuSi groups, indicating that the best angiogenic effect of mild heat-assisted CuSi NWs.

## 4 Discussion

As one-dimensional nanomaterials, NWs usually display unique optical, electronic, or mechanical properties due to the high transverse to longitudinal ratio structure and two-dimensional confinement, making them widely applied in multiple areas including tissue regeneration (Cao et al., 2018; Jones et al., 2018; Huang et al., 2021). Previous studies demonstrated that hydroxyapatite NWs could accelerate hemostasis and wound healing due to the excellent hydrophilicity, release of blood coagulation factor Ca^2+^, and flexibility to composite with other materials, while silver NWs showed a high-efficiency eradication rate against various bacteria including *E. coli* and *S. aureus*, and high potential as an antibacterial ingredient for wound dressing due to the sustained release of Ag ions (Sun et al., 2018; Wan et al., 2020; Zheng et al., 2022). However, none of these NWs possess antibacterial activity and skin-beneficial bioactivity simultaneously. Our proposal of CuSi NWs showed the advantages as high antibacterial efficiency and high bioactivity of angiogenesis were accomplished at the same time with the assistance of mild photothermal therapy. The *in vitro* antibacterial/cellular experiments and the *in vivo* animal experiments fully demonstrated that CuSi NWs might be a good candidate for the treatment of infected wounds.

The high antibacterial performance of CuSi NWs is mainly attributed to the combination of PTT and sustained Cu ions. Both of them were widely reported as broad-spectrum antibacterial strategies. However, their antibacterial mechanism is different. For photothermal antibacterial therapy, the photothermal agent is required to convert light to generating heat, thereby killing bacteria through the thermal effects including rupturing cell membranes, evaporating cell fluid evaporation, and destroying cellular protein/enzyme (Yang et al., 2017; Xu et al., 2019). NIR light is usually implemented in the process due to the good tissue penetration ability and high biosafety depending on the power density (Geng et al., 2018; Chen et al., 2020). Unlike PTT, Cu ions can kill bacteria by destroying the cell membrane/DNA through an electrostatic interaction or generating reactive oxygen species (ROS), and the efficiency is positively related to the concentration of Cu ions (Ning et al., 2015; Perdikaki et al., 2016; Slavin et al., 2017; Li et al., 2019). Since neither of these two methods is satisfactory as a single strategy in the treatment of bacteria-infected wounds due to concerning the possible negative effects on normal tissues by high temperature or high concentrations of Cu ions, recent investigations have elaborated that the combination of PTT and Cu ions might produce a “hot Cu ion” effect by improving the antibacterial efficiency and reducing the side effects of PPT and Cu ions when used alone (Xu et al., 2020; Xu et al., 2021). The “hot Cu ion” effect has been proven to be not only effective to common bacteria, but also the drug-resistance bacteria. A recent study exhibited that the “hot Cu ion” effect could even significantly inhibit the formation of biofilms due to the direct synergetic antibacterial effect of heat and Cu ions and the indirect pro-inflammatory effect by inducing macrophage towards M1 phenotype (Xu et al., 2021). However, the temperature used in this study is about 50°C, which is still far away from absolute safety (Xu et al., 2018; Fan et al., 2019). Notably, the temperature used in our study (45–47°C) has been proven safe for multiple tissues as a mild temperature in the application of PTT (Ma et al., 2020; Yang et al., 2020). Whereas the antibacterial efficacy of CuSi + NIR group is still high (∼99.35% against *S. aureus* and ∼94.19% against *E. coli in vitro*, and 92.51% against *S. aureus in vivo*), which is comparable and even better to other PPT combined strategies (Yuan et al., 2015; Li et al., 2017).

Apart from combating bacteria, promoting neovascularization is also important for the regeneration of injured skin. Numerous attempts have been made to improve the angiogenic ability of wound dressing and incorporation with angiogenic growth factors is one of the most direct and effective approaches (Song et al., 2021; Xie et al., 2023). However, the inherent disadvantages of proteins including the high cost, short half-lives, and low stability limit their clinical practice. Our previous studies demonstrated that silicate biomaterials usually possessed strong pro-vascularized ability including promoting proliferation, migration, and angiogenic gene expression of endothelial cells due to the sustained release of Si ions (Li and Chang, 2013; Zhou et al., 2018). Several silicate bioglasses or bioceramics and their composted materials have been developed as wound dressings with enhanced angiogenic ability for different wound healing including infected wounds, burn wounds, and diabetic wounds (Fan et al., 2022). More interestingly, by combining with the different metal elements, the bioactive of silicate materials could be further enhanced. For instance, Cu ions play a vital role in vascularization, which are mainly existed in the form of Cu-binding proteins in human body and show stimulatory effects on new vessel formation by activating several angiogenic pathways including the *HIF-VEGF* signaling pathway as Cu ions can stabilize the *HIF-1α* by preventing its degradation (Kong et al., 2014). The combination of Cu and Si ions may produce a synergistic effect on stimulating angiogenesis and facilitate the regeneration of injured tissues, which have been verified in different defect tissues such as bone, skin, and endometrium (Yang et al., 2021a; Yang et al., 2021b; Dong et al., 2022). Our present study research proved that CuSi NWs could significantly promote the expression of *VEGF*, *HIF-1α*, and *bFGF* in HUVECs and stimulated more neovascularization in the newly formed dermal tissue, which may also be ascribed to the synergetic angiogenic effect of Cu and Si ions.

Although we have successfully fabricated a new CuSi NW and demonstrated its potential therapeutic capacity in treating cutaneous infectious wounds *via* both *in vitro* and *in vivo* experiments. There are still some issues or limitations that need to be solved or improved in further studies. First, CuSi NWs suspension was applied in this study, which would precipitate after a while and may affect the final therapeutic effects. Composite material such as incorporating CuSi NWs into a commercial hydrogel matrix or an electrospinning film may be a better choice. Second, more biosafety analysis should be conducted since several potential risks were involved in this system including the utilized NIR light and possible nanotoxicity. Finally, the underlying mechanism of the enhanced wound healing ability of CuSi NWs should be explored as endothelial cell is only one of the cells that participate in wound repair and angiogenesis is also one of the factors that affect wound healing.

## 5 Conclusion

In summary, we proposed a NIR light-assisted approach to treat infected wounds based on the newly synthesized photothermal agent of CuSi NWs, which could not only effectively combat bacteria, but also promote angiogenesis and facilitate wound healing due to the sustained release of bioactive Cu and Si ions. Our results suggested that CuSi NWs have the potential for rapidly erasing bacterial infections and prominently promoting wound healing.

## Data Availability

The original contributions presented in the study are included in the article/supplementary material, further inquiries can be directed to the corresponding authors.
